# Comparison of anxiety-like and social behaviour in medaka and zebrafish

**DOI:** 10.1038/s41598-022-14978-1

**Published:** 2022-06-28

**Authors:** Tyrone Lucon-Xiccato, Felix Loosli, Francesca Conti, Nicholas S. Foulkes, Cristiano Bertolucci

**Affiliations:** 1 Department of Life Sciences and Biotechnology, University of Ferrara, Ferrara, Italy; 2 Institute of Biological and Chemical Systems, Biological Information Processing (IBCS-BIP), Karlsruhe Institute of Technology, Eggenstein-Leopoldshafen, Germany; 3 Department of Physiology, Faculty of Biology, University of Murcia, Murcia, Spain

**Keywords:** Animal behaviour, Animal physiology

## Abstract

The medaka, *Oryzias latipes*, is rapidly growing in importance as a model in behavioural research. However, our knowledge of its behaviour is still incomplete. In this study, we analysed the performance of medaka in 3 tests for anxiety-like behaviour (open-field test, scototaxis test, and diving test) and in 3 sociability tests (shoaling test with live stimuli, octagonal mirror test, and a modified shoaling test with mirror stimulus). The behavioural response of medaka was qualitatively similar to that observed in other teleosts in the open-field test (thigmotaxis), and in 2 sociability tests, the shoaling test and in the octagonal mirror test (attraction towards the social stimulus). In the remaining tests, medaka did not show typical anxiety (i.e., avoidance of light environments and preference for swimming at the bottom of the aquarium) and social responses (attraction towards the social stimulus). As a reference, we compared the behaviour of the medaka to that of a teleost species with well-studied behaviour, the zebrafish, tested under the same conditions. This interspecies comparison indicates several quantitative and qualitative differences across all tests, providing further evidence that the medaka responds differently to the experimental settings compared to other fish models.

## Introduction

In the last decade, a number of teleost species have become important laboratory models for research on behaviour, cognition, and related disorders^1,2^. One of these species, the medaka *Oryzias latipes*, offers a wide range of genetic tools to unravel the molecular, cellular and developmental mechanisms underlying behaviour^3–6^, and to conduct behavioural screenings of new drugs and xenobiotics^7–9^. The success of a model organism in these fields has often been enabled by the development of simple, standardised behavioural paradigms (e.g.,^10,11^). Despite the number of studies on medaka behaviour growing rapidly (e.g.,^12–14^), there are still relatively few standard behavioural tests that have been applied with this model. Here, we aim to investigate the possibility of studying medaka behaviour with tests developed for and widely-used in other teleosts.

The behavioural tests commonly adopted in fish models, and especially for the zebrafish, are mostly directed to measure anxiety-like behaviours. The open-field test is perhaps the most common of such tests^15–17^; it assesses the reaction of an individual to a novel, empty arena. Two other paradigms are routinely adopted to study anxiety-like behaviour in fish. The scototaxis test measures anxiety-like behaviour as the tendency to avoid a white, as opposed to a black sector of the experimental arena^18,19^, assuming that a fish would feel safer in the black sector whereby it is less visible to potential predators. The diving test measures anxiety-like behaviour in relation to the swimming depth of the fish: more anxious individuals usually tend to swim closer to the bottom^20,21^.

Many studies have also used tests based on the social behaviour of fish^22^. In the typical paradigm to measure shoaling tendency, the subjects are observed in a 3-chamber apparatus, in which an extremity contains a group of conspecifics as social stimulus (e.g.,^23–26^). The time spent by the subject in close proximity to the social stimulus is taken as a measure of sociability. Other versions of the sociability test involve the use of a mirror as stimulus^24,27,28^. If tested in a novel environment, the fish initially tend to show a strong affiliation response to their mirror image, which is probably perceived as a conspecific^24,27,28^.

The aforementioned tests may also be valid to investigate behaviour in medaka, and in some studies they have already been adopted (e.g.,^29–32^). However, an evaluation is first required because behavioural assessment in fish is subject to a number of potential problems (reviewed in ^17^). For example, Burns^15^ has demonstrated that the open-field test serves as a robust assay for the behaviour of guppies (*Poecilia reticulata*), but that other tests commonly used in this species are less robust. Other evidence indicates that a test might measure different behavioural traits in different species^33^. In addition, for fish, a number of studies have revealed how behavioural assessment can be altered by small changes in the experimental setting and the apparatus^34,35^. For example, studies in eastern mosquitofish, *Gambusia holbrooki*, and the Mediterranean killifish, *Aphanius fasciatus*, indicated that fish open-field behaviour is altered by the size of the experimental arena^36,37^.

The aforementioned issues call for an understanding of the performance of medaka in anxiety-like behaviour and sociability tests before developing models based on this species. In this study, we aimed to achieve this goal. We observed the behaviour of medaka in an open-field test (anxiety test 1), a scototaxis test (anxiety test 2), a diving test (anxiety test 3), a shoaling test (sociability test 1), a mirror test (sociability test 2), and a modified version of the shoaling test with mirror stimulus (sociability test 3). Importantly, we compared the performance of medaka with that of zebrafish, to understand whether a behavioural test has the same significance in these two species.

## Materials and methods

### Experimental fish

The medaka used in this study belonged to a wild-type strain (‘iCab’ strain) of the Karlsruhe Institute of Technology (KIT, Germany). This strain is derived from the Southern Japanese medaka population and is commonly used for basic research^12,38–40^. The experimental subjects were selected from a medaka population of approximately 500 individuals in the facility of the University of Ferrara. The population was maintained by regularly collecting fertilised eggs from the females and raising them in small aquaria following standard protocols. We also routinely moved groups of individuals between the various maintenance tanks in order to randomise the breeders.

The zebrafish were derived from a wild-type strain (‘Ariosto’) founded from fish bought at a local shop (N = 100) and maintained at the University of Ferrara’s facility since 2011. The population consists of approximately 1500 individuals and is maintained by routinely performing reproduction with standard procedures, using breeders randomly selected from the different tanks. In addition, we added 50–100 new zebrafish from the shop each year. We chose this population because many laboratories use similar strains of wild-type zebrafish for basic research^41^.

### Maintenance conditions and subject selection

Maintenance conditions were identical for both species to avoid confounding effects on behaviour. The fish were kept separate per species in groups of 80–100 individuals in standard 200 L glass aquaria. Using 24 aquaria, we maintained a pool of 16–18 groups of zebrafish and 6–8 groups of medaka from which we selected the individuals to be used in the experiments. Each aquarium was provided with mechanical and biological water filters. The temperature of the facility was maintained at 28 ± 1 °C by means of an automatic air conditioning system, thereby ensuring constant water temperature in the aquaria. The pH of the water in the facility was set to 8.0 and regularly checked in the aquaria (8.04 ± 0.13, mean ± standard deviation)^42^. Light-emitting diodes (LED) lamps placed on the ceiling of the facility provided illumination and were controlled by electronic timers to provide a 14 h light/10 h dark photoperiod. Fish received live (*Artemia salina* nauplii) and dry food ad libitum twice per day. At the end of the behavioural testing, each subject was transferred to post-experiment aquaria to avoid re-using them. These post-experiment aquaria had the same conditions as described above.

Overall, the study involved 114 medaka and 88 zebrafish. The subjects of each experiment were matched for size and age (10–12 months). For each experiment, we randomly selected the subjects from the groups in the maintenance tanks (N = 1–2 individuals from each aquarium). Moreover, each subject was tested in a single behavioural test. This subject allocation ensured high levels of biological replication for each behavioural test.

### Anxiety test 1: Open-field test

**Figure 1:**
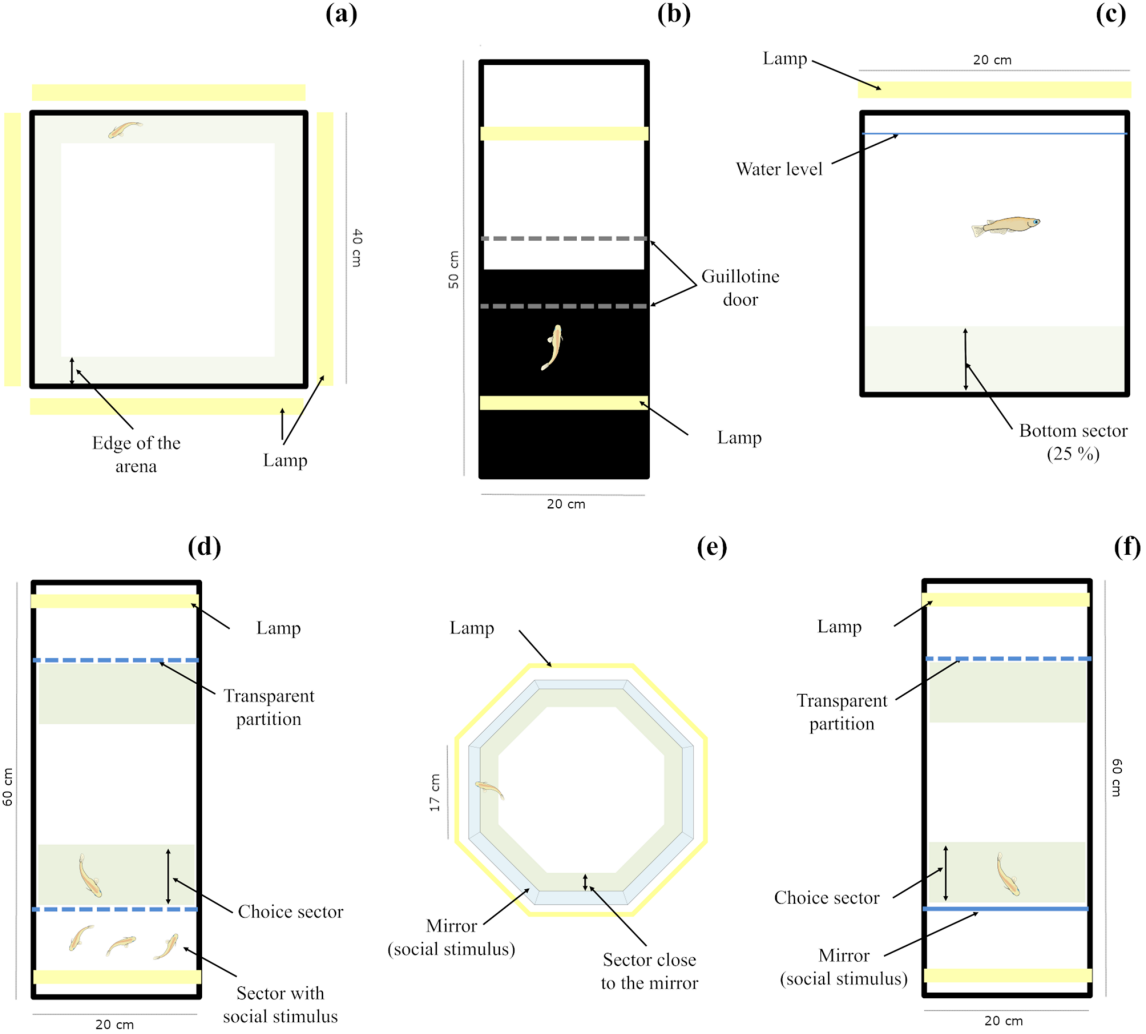
Schematic representations of the experimental apparatuses. (**a**) Open-field test; (**b**) scototaxis test; (**c**) diving test; (**d**) shoaling test; (**e**) octagonal mirror test; and (**f**) shoaling test modified with mirror stimulus. Green shadowing indicates sectors used to analyse fish behaviour; top views, except (**c**): side view.

In this experiment, we used 16 medaka and 8 zebrafish. The open-field test was conducted following a standard procedure^15,29,38,43^. We individually tested each subject in a 40 × 40 cm white plastic square arena, filled with 12 cm of water (Fig. 1a). The arena was positioned on a backlit table with infrared LED λ > 980 nm illumination (Noldus Information Technology, Netherlands). The table was kept in an experimental room with no illumination except for a warm-white LED strip (Superlight Technology Co. Ltd., Shenzhen, China) placed 1 m above the open-field arena. After collection from the maintenance tank, the subject was transported into the experimental room inside a small circular container. The container was gently emptied into the centre of the arena and the fish was let undisturbed to explore the novel environment. Initially, we tested 8 medaka and 8 zebrafish for 30 min, a sample size chosen based on earlier studies in zebrafish^44,45^. Then, we assayed 8 additional medaka with a longer testing time (4 h) in an attempt to observe any detectable habituation (see Results section). The subjects’ behaviour was analysed by means of a camera sensitive to infrared light (Basler Monochrome GigE camera, Germany; resolution: 1280 × 1024) placed 1 m above the open-field arena. The camera was connected to a computer running the EthoVision XT tracking software (Noldus Information Technology, the Netherlands), which recorded the position of the fish at 5 frames per second and used it to calculate 3 behavioural variables typically adopted in the open-field experiments. The first variable was thigmotaxis as time spent within 1 body length from the edge of the arena. This variable represented a measure of preference for the edge of the arena because it is based on a dichotomous spatial preference: during the entire test, the fish could either be, or not be at the edge. This approach is often used because a dichotomous preference is unlikely to be affected by confounding parameters such as the quantity of movement of the subjects. The second variable was the activity, measured as distance moved and the third variable represented another measure of activity calculated as the time spent moving within 1 body length/second as a threshold^29^. All the variables were saved in 1-min time bins to analyse the temporal pattern of behaviour.

### Anxiety test 2: Scototaxis test

We assayed 16 medaka and 16 zebrafish in the scototaxis test. The sample size was calculated based on the results of the first experiment using the software GPOWER version 3.1^46^. The procedure followed that of most studies in fish (e.g.,^19,47^). We tested each fish in a glass apparatus with rectangular base (50 cm × 20 cm, water depth: 12 cm; Fig. 1b). The apparatus’s walls and bottom were half white and half black, and each half was illuminated by a fluorescent white-light tube (15 W; Feilo Sylvania Germany GmbH, Germany). At the beginning of the test, we left the subject for 1 min for habituation in a central sector of the apparatus, between 2 guillotine doors. After this habituation, we lifted the guillotine doors and let the fish swim undisturbed in the apparatus for 30 min. The experiment was recorded with a camera (LEGRIA HF R38, Canon Inc., Japan) placed 1 m above the apparatus. The recordings were scored by an experimenter using a custom software (Ciclic timer) to calculate time spent in the white sector of the apparatus by each subject in 1-min time bins as the dependent variable. Considering that the fish’s spatial position could be only one of two possibilities (either in the white or black sector), our dependent variable provided a measure of preference for the white sector over the black sector, independent from other traits such as subjects’ swimming speed or activity.

### Anxiety test 3: Diving test

We observed 16 medaka and 16 zebrafish in a diving test using standard procedures^20,21^. Each subject was released into a small novel tank (20 cm × 20 cm) filled with 20 cm of water (Fig. 1c). Three walls of the apparatus were made by white plexiglass, whereas the remaining side was made of transparent plexiglass. The apparatus was illuminated by a fluorescent lamp (15 W; Feilo Sylvania Germany GmbH, Germany). A camera on a tripod recorded the behaviour of the fish from the lateral, transparent wall, for a period of 30 min. Using the EthoVision XT tracking software, we then tracked the position of the fish from the video recordings, calculating the dependent variable as the time spent swimming in the lower 25% of the apparatus in 1-min time bins. With this scoring system, the fish could only be either in the lower or in the upper sector of the apparatus; consequently, the dependent variable provided essentially a measure of preference for the lower sector over the upper sector.

### Sociability test 1: Shoaling test

The shoaling test was performed with 33 medaka and 15 zebrafish. As in prior studies (e.g.,^24^), the fish were recorded in a 3-chamber apparatus filled with 10 cm of water (60 cm × 20 cm × 20 cm; Fig. 1d). The central, larger chamber (30 cm × 20 cm) housed the subject for the entire test (30 min). One of the lateral chambers (15 cm × 20 cm) housed a group of conspecifics as social stimulus, whereas the other lateral chamber (15 cm × 20 cm) was left empty as a control. Each lateral chamber was lit by a fluorescent white-light tube (15 W; Feilo Sylvania Germany GmbH, Germany) to ensure high visibility of the stimuli. Initially, we tested 15 individual medaka and 15 zebrafish with 3 conspecifics as a social stimulus. Then, we assayed a further 18 medaka using 6 stimulus conspecifics in an attempt to understand the cause of an interspecific difference that was detected (see Results section). This additional test was performed the day after the main experiment to obtain comparable results. Using video recordings and the software Ciclic timer, the experimenter scored the time spent by subjects close to the conspecifics (within 2 body lengths) and using the same distance criterion, the time spent close to the empty sector. These measures were used to calculate a proportional index of shoaling preference as: time spent close to the stimulus/(time spent close to the stimulus + time spent close to the empty sector). This was necessary because the subjects could also spend time in the central, no-choice sector of the apparatus. In line with the approach used for the earlier tests, this dependent variable is a measure of spatial preference. However, the distance between the two choice sectors due to the central, no choice sector, might be problematic because fish with low activity might not notice the distant stimulus. Therefore, the experimenter also monitored a measure of activity consisting of counting switching’s made by each subject between the choice sectors (i.e., more active fish would switch more often between sectors). Data were collected in 1-min time bins.

### Sociability test 2: Octagonal mirror test

We tested 16 medaka and 16 zebrafish. Following prior studies in zebrafish and other teleosts^27,28,48,49^, we performed the mirror test using an octagonal apparatus with mirrored walls (Fig. 1e). The mirror covering each wall was 17 cm × 15 cm and the water in the apparatus was 8 cm deep. LED strips (Superlight Technology Co. Ltd., Shenzhen, China) placed around the apparatus’s walls ensured homogeneous illumination. The mirror apparatus was placed on the backlit table (infrared LED λ > 980 nm; Noldus Information Technology, the Netherlands) as previously described, to permit automatic tracking of the fish. The test procedure started with the release of an individual subject in the centre of the apparatus with the concomitant start of recording. The fish was then let undisturbed for the entire test time (30 min). As small social fish perceive their mirror image as a conspecific, they usually tend to swim in proximity of the mirror. The tracking software (EthoVision XT, Noldus Information Technology, the Netherlands) therefore calculated the time spent by each subject within 1 body length from the mirror (in 1-min time bins), which is essentially a measure of spatial preference between two sectors, in line with the variables collected in the previous experiments.

### Sociability test 3: Modified shoaling test with mirror stimulus

We performed this experiment to interpret contrasting results observed between sociability tests 1 and 2 (see Results section). The procedure and the apparatus were identical to those described in the sociability test 1 (shoaling test). However, we substituted the live stimuli with a mirror as in Cattelan et al.^24^ (Fig. 1e). We tested 17 medaka and 17 zebrafish.

### Statistical analysis

Statistical tests were performed using R version 3.2.2 (The R Foundation for Statistical Computing, Vienna, Austria, http://www.r-project.org). The tests were 2-tailed and the threshold for significance was P = 0.05. The main analysis for each dependent variable collected was performed with a repeated measures ANOVA. The model included species (medaka versus zebrafish) as between-subjects effect and testing time (i.e., 30 1-bin time bins) as within-subject factor. Time spent close to the mirror in the sociability test 2 was log transformed before the analysis to meet model assumptions. In the anxiety test 2, the sociability test 1, and the sociability test 3, the fish were exposed to a dichotomous choice between sectors of the apparatus with equal size (i.e., respectively: white versus black sector; sector close to the shoal versus sector close to the empty chamber; and sector close to the mirror versus sector close to the empty chamber). Therefore, we also performed a one-sample t-test on the dependent variable of the medaka (calculated over the entire testing time) to evaluate the presence of a significant preference for the relevant sector of the apparatus.

### Ethics declarations

Experiments were conducted in accordance with Italian law (Italy, D.L. 4 Marzo 2014, n. 26). The Ethical Committee of University of Ferrara reviewed and approved the experimental procedures (CB/01-2019). No physical invasive manipulations were performed on the fish during the experiments and no fish showed sign of distress. At the end of the experiments, all subjects were returned to the stock maintenance tanks.

## Results

### Anxiety test 1: Open-field test

**Figure 2:**
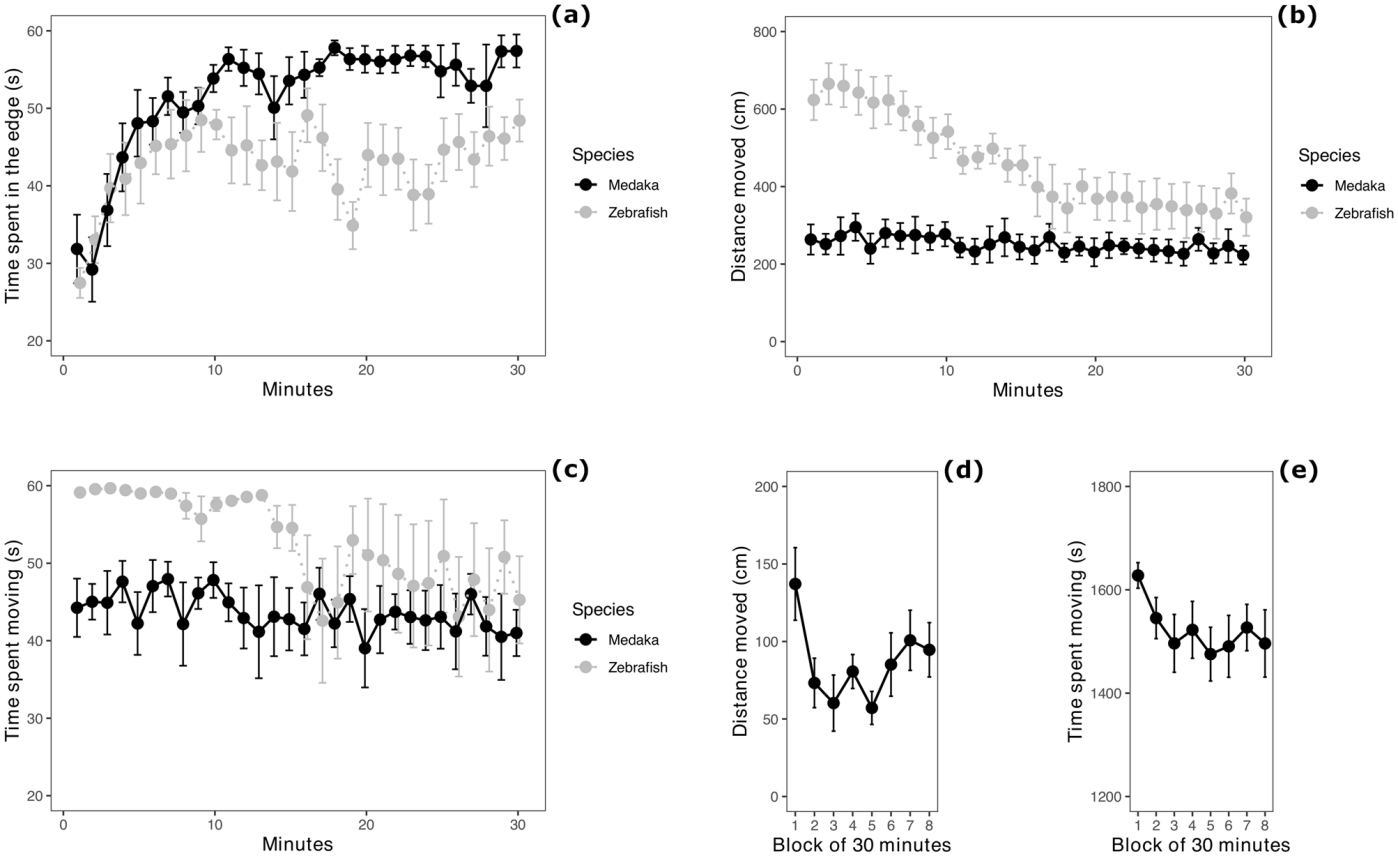
Open-field tests. (**a**) Thigmotaxis measured as time spent close to the edges of the arena, divided in 1-min bins; (**b**) activity measured as distance moved, divided in 1-min bins; (**c**) activity measured as time spent moving, divided in 1-min bins; (**d**) activity measured as distance moved in the medaka tested with the extended testing time (4 h), divided in 30-min bins to allow comparison between standard testing time (30 min) and extended testing time; (**e**) activity measured as time spent moving in the medaka tested with the extended testing time (4 h), divided in 30-min bins to allow comparison between standard testing time (30 min) and extended testing time. Data points and error bars represent means and standard errors, respectively.

The medaka spent most of the time in proximity of the edges of the arena (mean ± standard deviation: 51.66 ± 10.55 s/min). The analysis revealed that the medaka spent more time at the edge of the arena compared to the zebrafish (zebrafish: 42.93 ± 11.58 s/min; F_1,14_ = 7.831, P = 0.014; Fig. 2a). This difference was modulated by testing time (species × testing time interaction: F_1,462_ = 21.625, P < 0.001; Fig. 2a). There was also a significant general increase in the time spent at the edge of the arena (main effect of testing time: F_1,462_ = 74.033, P < 0.001).

The analysis of distance moved revealed a significant difference between species (F_1,14_ = 20.058, P < 0.001): medaka (distance moved, mean ± standard deviation: 251.28 ± 92.48 cm/min) were less active compared to zebrafish (distance moved: 459.98 ± 189.58 cm/min; Fig. 2b). There was also a significant main effect of time (F_1,462_ = 192.870, P < 0.001), modulated by a decrease in activity in zebrafish (species × time interaction: F_1,462_ = 123.981, P < 0.001; Fig. 2b). The analysis of time spent moving showed a pattern of activity comparable to that of distance moved (Fig. 2c): medaka were overall less active than zebrafish (F_1,14_ = 5.562, P = 0.033); there was a significant main effect of testing time (F_1,462_ = 43.839, P < 0.001), but this was mostly due to zebrafish (F_1,462_ = 16.233, P < 0.001; Fig. 2c).

The medaka tested for a longer period (4 h) showed a more marked decrease in activity across 30 min blocks of time (distance moved: F_7,49_ = 2.296, P = 0.042; time spent moving: F_7,49_ = 2.500, P = 0.028; Fig. 2d,e).

### Anxiety test 2: Scototaxis test

**Figure 3:**
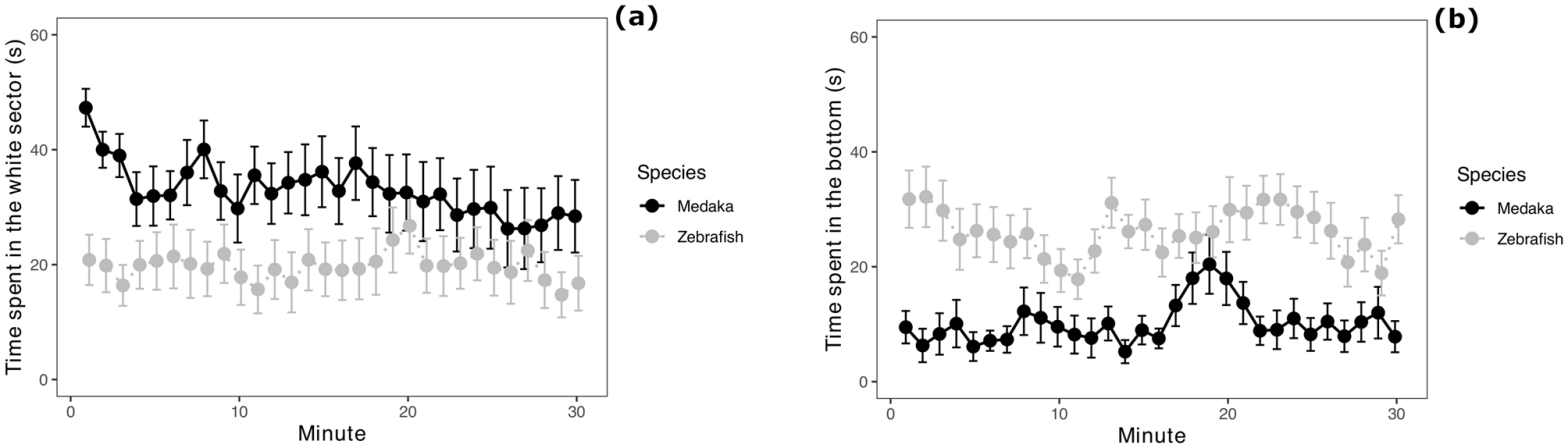
Scototaxis test and the diving test. (**a**) Time spent by subjects in the white sector of the scototaxis apparatus, divided in 1-min bins; (**b**) time spent by subjects in the bottom sector (25% of the area) of the diving test apparatus, divided in 1-min bins. Data points and error bars represents means and standard errors, respectively.

Medaka did not display avoidance of the white sector of the apparatus (one-sample t-test: t_15_ = 0.768, P = 0.454; time spent in the white sector, mean ± standard deviation: 33.05 ± 23.04 s/min). The analysis revealed that the medaka spent more time compared to the zebrafish in the white sector (zebrafish: 19.69 ± 19.32 s/min; F_1,30_ = 6.433, P = 0.017; Fig. 3a). There was also temporal variation in the time spent in the white sector of the apparatus (F_1,926_ = 11.783, P < 0.001), due to the medaka’s decrease in this variable (F_1,926_ = 10.203, P = 0.001; Fig. 3a).

### Anxiety test 3: Diving test

The medaka did not display a marked preference for the bottom part of the apparatus (time spent in the bottom, mean ± standard deviation: 10.15 ± 13.56 s/min). Zebrafish spent significant more time at the bottom of the apparatus compared to medaka (26.13 ± 17.71 s/min; F_1,30_ = 20.458, P < 0.001; Fig. 3b). The swimming depth of the fish did not vary across testing time (F_1,925_ = 0.771, P = 0.380) and the species × time interaction was not significant (F_1,925_ = 2.170, P = 0.141).

### Sociability test 1: Shoaling test

**Figure 4:**
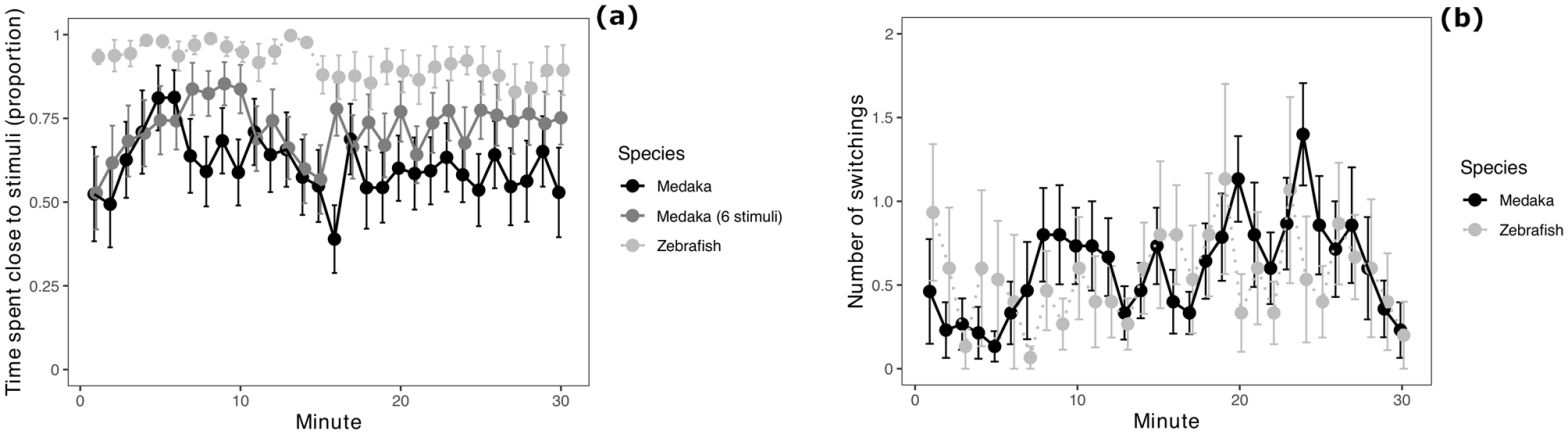
Shoaling test. (**a**) time spent by subjects in the sector close to the shoal of conspecifics in the shoaling test, divided in 1-min bins; (**b**) number of switchings between the 2 choice sectors performed by subjects in the shoaling test, divided in 1-min bins. Data points and error bars represents means and standard errors, respectively.

Medaka demonstrated a tendency to spend more time in the sector of the apparatus close to the social stimulus of 3 conspecifics (one-sample t-test: t_14_ = 1.981, P = 0.068; proportion of time spent close to the conspecifics, mean ± standard deviation: 0.61 ± 0.41). However, the preference displayed by zebrafish was significantly greater compared to medaka (zebrafish: 0.92 ± 0.21; F_1,28_ = 23.249, P < 0.001; Fig. 4a). There was also a significant effect of time (F_1,855_ = 9.855, P = 0.002), but no significant species × time interaction (F_1,855_ = 0.088, P = 0.768).

In the analysis of the number of times that the subject switched between the choice sectors, there was a significant effect of time (F_1,855_ = 4.716, P = 0.030; Fig. 4b). There was no significant effect of species (F_1,28_ = 0.073, P = 0.790) and no significant species × time interaction (F_1,855_ = 1.348, P = 0.246).

In the subsample of medaka tested with an increased number of social stimuli (6 conspecifics), the sociability was still below the level of zebrafish (Fig. 4a).

### Sociability test 2: Octagonal mirror test

**Figure 5:**
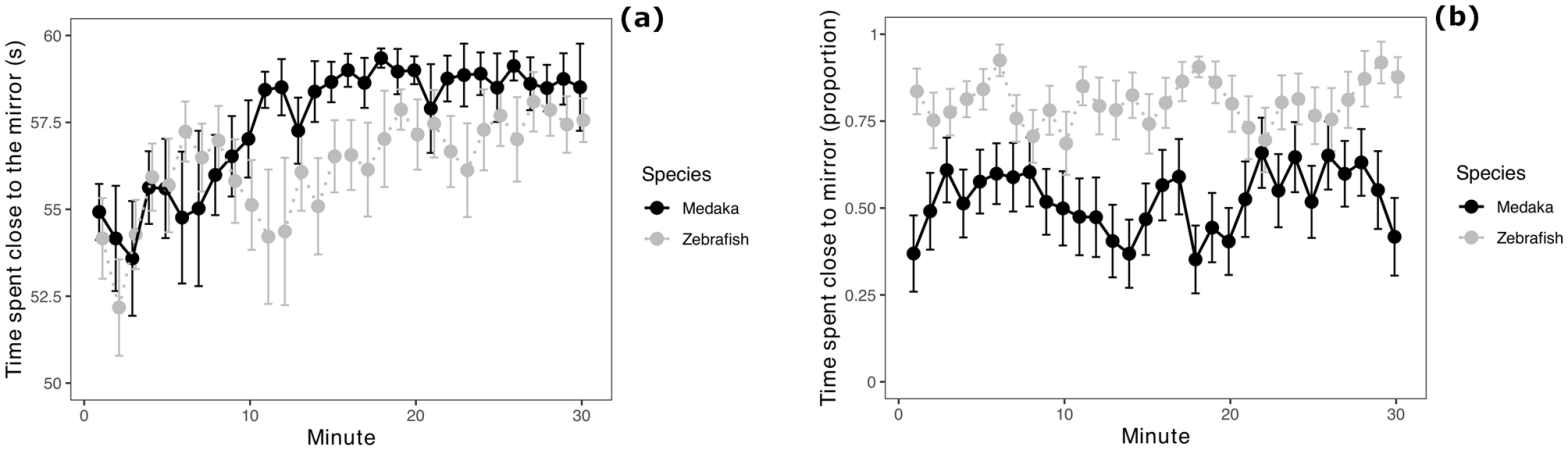
Results of the octagonal mirror test and the modified shoaling test with mirror stimulus. (**a**) Time spent by subjects within 1 body length from the mirror walls in the octagonal mirror test, divided in 1-min bins; (**b**) proportion of time spent by subjects in the sector close to the mirror in the modified shoaling test, divided in 1-min bins. Data points and error bars represents means and standard errors, respectively.

The medaka spent most of the testing time in close proximity of the mirror (mean ± standard deviation: 57.53 ± 4.42 s/min). The analysis revealed a marginal difference between the medaka and the zebrafish (F_1,30_ = 3.931, P = 0.057). The medaka showed greater sociability compared to zebrafish after approximately 10 min of testing (species × time interaction: F_1,926_ = 9.778, P = 0.001; Fig. 5a). The social attraction towards the mirror image increased significantly across testing time (F_1,926_ = 151.472, P < 0.001).

### Sociability test 3: Modified shoaling test with mirror stimulus

Medaka did not show a significant preference for the mirror stimulus (one-sample t-test: t_16_ = 0.472, P = 0.643; mean ± standard deviation: 0.52 ± 0.42). The preference for the mirror stimulus over the empty sector of the apparatus was significantly greater in zebrafish compared to medaka (zebrafish: 0.80 ± 0.30; F_1,32_ = 34.384, P < 0.001; Fig. 5b). There was no significant effect of time (F_1,975_ = 1.919, P = 0.166), nor significant species × time interaction (F_1,975_ = 0.092, P = 0.762).

## Discussion

In this study, we evaluated the behaviour of the medaka in a select number of standard anxiety and sociability tests developed for other teleost species: the open-field test, the scototaxis test, the diving test, the shoaling test, the octagonal mirror test, and a modified shoaling test with mirror stimulus. Individual medaka fish showed distinct and measurable behaviours in all the tests. However, in some tests, medaka showed a species-specific behaviour that differed from that of other teleost models. We thus found remarkable differences between the scores of the medaka and those of zebrafish, which we used as a reference species.

### Behaviour of medaka in the anxiety tests

In the anxiety test 1, the open-field test, the medaka showed the typical thigmotaxis response of other fish species, that is they avoided the centre of the apparatus and rather preferred to swim close to the edges. The explanation for thigmotactic behaviour in a novel environment is that the fish perceive the centre of the arena as more exposed and therefore riskier. This represents a logical behavioural response because the medaka is predated by almost 20 species in its natural environment^50^. We therefore expect the medaka to display behavioural patterns that minimise the risk of detection by predators, such as thigmotaxis. Interestingly, in medaka the thigmotaxis was not stable across testing time, but underwent a marked increase at the beginning of the experiment. This could be due to an initial habituation to the apparatus, although data of the following variables do not support the presence of rapid habituation in this species. Therefore, it is possible that when the medaka is placed in a novel environment, it initially explores the entire arena and only later on, expresses a spatial preference for the edges. Regarding the research application of the test, thigmotaxis is typically considered as an indicator of anxiety^19,51^, suggesting that the open-field test can be used to assess this behaviour in the medaka.

Additional parameters indicating anxiety-like behaviour in the open-field test are the distance moved by the subjects and the time spent moving (2 measures of activity), and especially, the change of these parameters over time (habituation). Individuals with high anxiety-like behaviour levels usually display lowered activity^16^ and slow habituation^52^. The activity of the medaka in the open-field test was successfully detected by our system but unexpectedly, it was constant across the entire testing time (30 min), denoting absence of habituation. We therefore performed an additional test with an extended duration (4 h) in a group of new subjects. In this second case, we did observe evidence for habituation as a reduction of activity over time. We conclude that the medaka displayed habituation to the open-field, but relatively slowly. The rate of habituation could be determined by anxiety but also by non-associative learning^53^. A hypothesis that medaka might display reduced learning does not appear plausible: this non-associative learning capacity is indeed broadly encountered in animals and even in non-neural organisms such as moulds^54^. Therefore, it is more likely that the anxiety of medaka during the test was elevated, causing slower habituation to the novel environment. Considered as a whole, the results of anxiety test 1 suggest that it is possible to assess anxiety-like behaviour in medaka with the open-field test, and that thigmotaxis is probably the most effective practical parameter for this purpose compared to habituation. Habituation can perhaps be measured with different protocols in medaka, such as those that require repeated exposures to the same arena^30,55^ or those involving different types of stimuli^56^.

In the remaining two anxiety tests, the behaviour of the medaka was distinct from that of other teleosts^19^. In the anxiety test 2, the scototaxis test, medaka spent approximately half of their time in the white sector of the apparatus. Most fish avoid the white sector of the apparatus and spend most of the time in the black sector, a response that is often attributed to seeking refuge in the environmental zone with the darkest substrate. As mentioned before, medaka are subjected to intense predation risk in their natural environment^50^. The lack of a clear scototaxis response is therefore unexpected and unlikely to be explained by the absence of antipredator behaviour. We speculate that the medaka does not use scototaxis as refuge seeking behaviour to deal with predation risk, possibly because they have a body colouration that reduces their visibility even over clear substrates in their natural habitat. Therefore, alternative refuge seeking behaviours might be more important for medaka. For example, an analysis of the distribution of medaka in natural rivers has indicated that they tend to seek refuge by exploiting cover and vegetation rather than different substrates^57^. Since medaka do not show the expected response to the scototaxis test, this test might not be suitable for assessing anxiety-like behaviour in this species.

In the anxiety test 3 (diving test), anxiety-like behaviour is measured as the preference to swim at the bottom of the test tank. This swimming preference was not observed in the medaka. It is therefore difficult to recommend use of the diving test to measure medaka anxiety-like behaviour due to reduced avoidance of the top of the apparatus. This does not automatically exclude that the medaka failed to display anxiety during the test. As discussed for the scototaxis test, it is more likely that the medaka reacted differently to the apparatus compared to what is observed in other species. For example, field sampling of wild medaka has revealed a preference for shallow water environments (20–30 cm)^57,58^. Therefore, medaka might not seek deeper waters as a response to dangerous situations, such as the novel environment of anxiety test 3. In conclusion, the absence of the diving response suggests that this test is not well suited for the medaka.

### Behaviour of medaka in the sociability tests

In our sociability test 1, medaka responded to the social stimuli with attraction (although only marginally significant), spending approximately 60% of their time close to the social group in the live stimulus test. Notably, the medaka showed relative high levels of activity in the apparatus, switching between choice sectors almost every minute, indicating that they were likely aware of both stimuli. When tested with the octagonal mirror test, medaka spent approximately 95% of their time close to the mirror image, indicating that they misperceived it as a social companion. These findings suggest that qualitatively, medaka shows the typical response of social teleost fish to these behavioural tests, although with large quantitative variability. The qualitative similarity is not surprising considering that the presence of social behaviour in this species has been highlighted in several previous studies^31,59,60^. It was also found that medaka are capable of sophisticated social interactions based on recognition of individual group mates^14^. Social behaviour tests are therefore applicable to medaka. In the context in which we performed the experiments (i.e., in a novel environment), social attraction likely serves as a response to uncertainty^61^. This may explain the absence of scototaxis and diving response observed in the anxiety tests. Indeed, it is tempting to speculate that medaka might rely mostly on social behaviour as a strategy to cope with danger.

Interestingly, the social attraction of the medaka was much greater towards the mirror stimulus compared to the live conspecific stimuli. The use of larger stimulus shoal (from 3 to 6 individuals) only slightly increased the social attraction towards live conspecifics. The increased response to the mirror as compared to the live stimuli is unexpected for two reasons. Firstly, fish usually prefer larger shoals in this type of tests^34,62,63^, and the live stimuli consisted of multiple fish whereas the mirror stimulus represented a single fish. Secondly, the live fish were expected to provide a more realistic stimulus^24^. Therefore, considering that the medaka showed a stronger avoidance of open spaces in the anxiety test 1 (i.e., high thigmotaxis behaviour), we hypothesised that such behaviour might account for the apparent increase in social preference (time spent close to the mirror at the edges of the arena) in sociability test 2 compared to sociability test 1. To test this potential explanation, we performed a third sociability test that employed a mirror stimulus (as in sociability test 2) in an apparatus with 3 chambers (as in sociability test 1). In this third sociability experiment, we found that the medaka sociability was more similar to that expressed with the live stimuli (sociability test 1) compared to the octagonal mirror test (sociability test 2). We conclude that the high sociability observed in the octagonal mirror test was due to a tendency of the medaka to avoid the centre of the arena, at least in part independently from the presence of the social stimulus. Overall, medaka clearly show a social behaviour that can be analysed in standardised behavioural tests. However, the score of the test is strongly affected by the shape of the testing apparatus due to the influence of other behavioural traits. This issue should be considered carefully.

### Behaviour of medaka compared with zebrafish

We found several behavioural differences between medaka and our reference species, the zebrafish. In anxiety test 1, the thigmotaxis of the medaka was more pronounced compared to the zebrafish. This interspecific difference emerged after approximately 10 min of testing, coincidently with other behavioural changes in the zebrafish (i.e., activity). Apparently, both species initially increased thigmotaxis at a similar rate, but then the zebrafish reached a plateau for this behaviour earlier (Fig. 2a). The reason for this trend is unclear. It may be that the medaka take longer to habituate to the novel arena, as also suggested by the results of habituation in activity. Alternatively, the medaka might take longer to explore the novel environment at the beginning of the test with respect to the zebrafish; interspecific differences in exploratory behaviour have indeed been observed even among closely related fish species^64^.

The activity of the medaka in anxiety test 1 was significantly lower compared to zebrafish. The effect was more evident at the beginning of the test. This might indicate different anxiety responses in the two species^16^, in line with the data of thigmotaxis. However, our experiment cannot completely exclude that the medaka simply exhibited a lower swimming capacity. Indeed, in a recent study with a swimming tunnel, we found that the maximum speed of this zebrafish strain was higher compared to that of the medaka^65^. Importantly, in some behavioural tests, the low activity of medaka can be problematic and hamper data collection^66^. However, it is important to note that the main behavioural parameters measured in our other tests were mostly unaffected by activity because they relied on dichotomous spatial preferences. Furthermore, in the sociability test 1 which requires a choice between 3 sectors, medaka and zebrafish displayed similar activity (i.e., number of switchings between sectors). The analysis of activity in the open-field also indicated that the habituation of zebrafish was much faster with respect to that displayed by medaka, only after a 30 min interval of testing. This makes it impractical to use medaka as a model for studies focused on this type of habituation behaviour.

In anxiety test 2 and anxiety test 3, the comparison between medaka and zebrafish is problematic. While the quantitative analysis suggested lower anxiety of medaka in both tests, we hypothesise that the difference was more likely qualitative. The medaka did not show scototaxis or the diving behaviour, suggesting that they did not respond to the tests. This might be inherently related to the fact that these experimental conditions are not equally well suited to measure the same behaviour in both species. To the best of our knowledge, the scototaxis and the diving tests have been specifically designed for the zebrafish, although it is known that other species show similar anxiety-like behaviour in the same assays (e.g.,^19^). Future studies should aim to implement modified versions of these assays, tailored better to the behaviour of medaka.

The apparent absence of anxiety-like behaviour in medaka as indicated by the anxiety tests 2 and 3 might result in some serious interpretative errors. For example, an investigation based on the scototaxis test or the diving test alone may lead researchers to conclude that medaka has lower levels of anxiety-like behaviour compared to the zebrafish. This erroneous conclusion disregards the fact that medaka did not respond to the tests. A different conclusion can be drawn, based on the results of the anxiety test 1 alone, whereby the medaka demonstrated greater anxiety-like behaviour compared to the zebrafish in the open-field test. It is therefore important to use experimental paradigms that elicit an appropriate behaviour in the tested species to avoid interpretative errors. Another factor to consider is that the medaka appeared generally less anxious in the two tests performed in a compact test tank, the scototaxis and the diving test, compared to the test performed in a large open arena (the open-field test). It is therefore possible that medaka anxiety-like behaviour is increased in open arenas.

Regarding sociability tests, results of the interspecific comparison were mixed. In the shoaling test (sociability test 1) and the modified mirror test (sociability test 3), medaka’s attraction to live social companions was lower compared to that of zebrafish. In the octagonal mirror test (sociability test 2), the opposite pattern was observed, with medaka being more attracted to their mirror image than zebrafish. Based on the results of sociability test 3, we conclude that this was due to the strong thigmotaxis response exhibited by the medaka in open-field arenas, which could also be interpreted as social attraction in the octagonal mirror test. We cannot exclude that, at least in part, the two species responded differently to the social stimuli because the mirror image provides only visual information and lacks olfactory cues. In prior studies with contrasting cues, zebrafish has shown a strong dependence on olfactory cues for identifying social companions^67^, while medaka appear highly attuned to visual details of their group mates^14,68^.

Overall, our anxiety tests suggest that medaka’s anxiety-like behaviour can be measured reliably by the thigmotaxis behaviour. However, for tests based on activity, habituation, scototaxis, and diving behaviour, the zebrafish might be a more appropriate model. Sociability represents an interesting behaviour to exploit in medaka research, however, attention should be given to the effects of the experimental setting on this behaviour. Regarding tests in open-field arenas, while sociability can be measured reliably in zebrafish, these tests may well be unsuitable for medaka. It is worth noting that medaka and zebrafish have had a long, independent evolutionary history and are native of different habitats. Therefore, these behavioural differences may well be related to species adaptation. Comparative studies including more diverse teleost species will be required to test this hypothesis.

## Conclusions

The medaka is becoming well established as a laboratory model to investigate the molecular and genetic basis of behaviour and its dysfunction. For all these applications, it is critical to adopt reliable and standard behavioural tests. The results of our comparative study support the use of some standard behavioural tests to assess anxiety-like behaviour and sociability in medaka. However, other tests yield qualitatively different results from that of other fish species and are potentially less suitable for medaka. This underscores the necessity to consider species-specific responses in the development of paradigms for examining behavioural traits.

## Data Availability

Data are included as Supplementary Information.
